# Effects of partial silage replacement with corn stover pellets on the rumen microbiota and serum metabolome of breeding cows

**DOI:** 10.3389/fmicb.2025.1533851

**Published:** 2025-02-25

**Authors:** Chenyue Jiao, Changze Cui, Youpeng Qi, Meixian Zhang, Pengcheng Zhao, Shaopeng Chen, Xiangyan Wang, Jiang Hu, Bingang Shi, Ting Liu, Zhidong Zhao, Fangfang Zhao

**Affiliations:** ^1^Gansu Key Laboratory of Herbivorous Animal Biotechnology, College of Animal Science and Technology, Gansu Agricultural University, Lanzhou, China; ^2^Linxia Beef Cattle Industry Development Research Institute, Linxia, China

**Keywords:** Simmental breeding cow, microorganisms, amino acid metabolism, stalk, green fodder supplementation

## Abstract

**Introduction:**

Straw pellet ration replacing part of silage is of great significance for farmers to save farming costs and solve the lack of feed resources. A comprehensive analysis of rumen microbial and serum metabolite compositions is conducted to promote the development of the modern breeding cows-feeding industry.

**Methods:**

In this study, 18 healthy 2-year-old Simmental breeding cows weighing 550 ± 20 kg were selected and randomly divided into two groups. They were fed under the same feeding conditions for 70 days, of which 8 in the control (CON) group were fed 65% roughage (100% silage) + 35% concentrate, and 10 in the treatment (TRT) group were fed 65% roughage (50% corn stover pellets +50% silage) + 35% concentrate, and milk quality, serum immunity indexes, serum metabolomes, rumen fermentation parameters, rumen Microorganisms.

**Results:**

The results showed that there was no significant difference in production performance between the two groups of breeding cows fed hay and Corn stover pellet feed (*p* < 0.05); Immunoglobulin A (IgA) was significantly higher in TRT compared to CON (*p* < 0.05), and there was no significant difference in Immunoglobulin G (IgG) and Immunoglobulin M (IgM) between the two groups (*p >* 0.05); a total of 92 differential metabolites were screened out in the serum metabolomics analysis, among them, L-valine, L-leucine, L-arginine, L-cysteine, L-tyrosine, and L-tryptophan were up-regulated; In rumen fermentation parameters there was no significant difference between CON and TRT in rumen pH, rumen ammonia nitrogen (NH_3_-N) content, rumen Acetic/Propionic concentration (*p >* 0.05), and the concentration of Acetic, Propionic, butyric and Total volatile fatty acids (TVFA) in CON was significantly lower than that in TRT (*p* < 0.05). Among the rumen microorganisms, the dominant groups were Thick-walled Firmicutes, Bacteroidota, *Prevotella* and *Ruminalococcus*. In the correlation analysis between rumen fermentation parameters and rumen microorganisms, Propionic and TVFA showed a significant positive correlation with *Prevotella* (*p* < 0.05), butyric showed a highly significant positive correlation with *Prevotella* (*p* < 0.01), and propionic butyric, and TVFA showed a positive correlation with *Bacteroides* (*p* < 0.05); L-cysteine was significantly positively correlated with *Prevotella* and *Anaeroplasma* (*p* < 0.05) and *Eubaterium* in rumen microbial-serum metabolite correlation analysis (*p* < 0.01).

**Conclusion:**

The microbial and metabolomic analyses provide us with essential data support to further provide a scientific basis for breeding cows feeding through the feeding pattern of straw pellets instead of silage, which will help breeding cows farming in future research.

## 1 Introduction

With the development of China’s livestock industry, the female cattle breeding industry occupies an important position in the national economy. However, China’s breeding cows breeding industry faces many challenges, especially in China’s Northwest region; due to geographical constraints, farmers can only make a small amount of corn to make silage to feed breeding cows; however, the shortage of feed resources, feeding costs and nutritional metabolism of disease, there will be a weak body, poor immunity, loss of appetite and other phenomena, and may even lead to endometritis, abomasum displacement, mastitis, and other obstetrical disorders (Ruegg and Milton, 1995; Gillund et al., 2001; Berry et al., 2007). These problems affect the health and performance of heifers and constrain the sustainable development of the industry. Therefore, finding efficient feed substitutes and further research on the mechanism of nutrient metabolism in heifers are key issues in the cows farming industry.

Globally, grass availability is a key constraint to beef cattle production as the market for beef cattle continues to expand (Zhang et al., 2022), moreover, the production of corn silage is insufficient in some areas of China, and its application in beef cattle farming is not popular; therefore, we used non-traditional feed resources as a significant component in beef cattle production to evaluate their benefits in beef cattle farming (Saunders et al., 2015). Ruminants primarily feed on forage, corn stalks, and grains, each with varying nutritional values. The formulated feed is designed to optimize nutrient absorption in breeding cows, thereby enhancing their immunity and preventing common nutritional deficiencies. China’s corn stover accounts for nearly one-fifth of the world’s stover resources, with a large amount of stover-type poor-quality roughage resources that are not yet fully utilized (Zhuang et al., 2020). Farmers in China often burn most of the straw directly, and in India, much of the post-harvest residue is burned in the fields, releasing large amounts of pollutants (Bhuvaneshwari et al., 2019). High straw yield and high crude fiber content can be used for straw feed treatment processing. Pelleting is an effective technology. After crushing, high temperature and pressure can kill parasite eggs and pathogenic bacteria and improve palatability, intake, digestibility, feed conversion rate, and livestock production performance (Zhang et al., 2019; Trabi et al., 2019). Some studies have shown that straw pelletization as complete pellet feed is an innovative feed technology that has gained tremendous popularity over the past few years due to its ease of feeding management, minor storage space requirement, and easy transportation. Domestic and international attention has been paid to the resources of straw, and straw modernization is an effective way to solve the waste of straw resources and the demand for animal feed (Adesogan et al., 2019). There is a high attention paid to straw resources at home and abroad. Straw feed is an effective way to solve the waste of straw resources and the demand for animal feed (Chen L. et al., 2017; Blanco et al., 2015). The shift to straw forage is not only an effective solution to the disposal of agricultural waste but also improves the economic efficiency of cows, reduces feed options, and ensures uniform nutritional delivery to the animals. Although silage is a common method to preserve fresh feeds such as whole corn, alfalfa and pasture oats, which can retain the nutrients in the raw materials and improve their quality and palatability (Li et al., 2015; Xu et al., 2020), silage is complicated to prepare and store, and is subject to seasonal and geographical limitations, in contrast, corn stover pellet feed has the advantages of simple preparation, convenient storage of balanced nutrition, and economic benefits, so corn stover Therefore, corn stover pellet feed is an ideal choice to replace traditional silage, and therefore has a broad application prospect in the breeding of cows.

The rumen is an important part of the digestion and absorption of nutrients in ruminants, with a highly stable and extremely complex micro-ecosystem, mainly composed of bacteria, protozoa, archaea and fungi (Guo et al., 2020). This unique microbial ecosystem leads to the development of a symbiosis between host and rumen microbial community composition that can provide about 70% of the energy for ruminants (Anderson et al., 2017; Rosenberg and Zilber-Rosenberg, 2018). Whereas diet composition and structure have important effects on rumen microbes, feeding pellet size or dietary physically effective fiber are important influences on masticatory activity, intestinal fiber and starch digestibility, and rumen pH (Zhao et al., 2011). In studies with lambs, pelleted native grass forage improved meat quality and increased animal performance (Bu et al., 2021). Roughage type and physical state are important factors influencing rumen fermentation and microflora (Wang et al., 2015). Animals on grain or grass-based diets have lower bacterial diversity (Xue et al., 2020; Ellison et al., 2014), and high feeds are beneficial to certain microorganisms, such as the phylum Thicket and Ascomycota (Khafipour et al., 2009). Ruminal microorganisms can communicate with the host through various metabolite libraries (Cui et al., 2020; Agus et al., 2021) and can directly influence the serum metabolome (Tanes et al., 2021). 16S rRNA sequencing is a well-tested, rapid and cost-effective method for analyzing differential abundance of microbial communities and correlation with environmental factors (Jami et al., 2013; Henderson et al., 2015). Metabolomics is the study of the types and quantities of endogenous small-molecule metabolites (<1,000 Da) and their patterns of change in cells, tissues, or organisms under specific physiological states of organisms, thus reflecting the functional state of biological systems (Cui et al., 2018). Liquid chromatography-mass spectroscopy (LC–MS) is commonly used, which not only avoids the need for complex sample pre-treatment procedures, but also offers a wider detection range and higher resolution and sensitivity, identification and quantification of numerous metabolites, and faster detection speeds (Ni et al., 2019). Blood metabolites are important indicators of the nutritional and physiological status of an animal, reflecting the health of certain tissues and organs of the animal (Chen et al., 2015). Mining data for potential information to identify key metabolites. Therefore, it is necessary to investigate the effects of feeding straw pellets in place of some silage on rumen microbes and serum differentials in postpartum breeding cows.

To the best of our knowledge, there is less literature on the use of corn stover pellet feed to replace 50% silage in feeding postpartum breeding cows, so the objective of this study was to find out the effects of feeding two different feeds on milk quality, serum metabolism and immunity indexes, rumen fermentation parameters and rumen microorganisms of breeding cows. The potential value of corn stover pellet feed was explored to further improve the scientific feeding of postpartum breeding cows and make a basis for industrial development in Northwest China.

## 2 Materials and methods

### 2.1 Test animals and experimental design

All animal experiments, including experimental design and feeding management, were approved by the Animal Ethics Committee of Gansu Agricultural University (Approval No. GSAU-Eth-AST-2023-034). This experiment was conducted from February 25 to May 15, 2024 at Shengze Agricultural and Animal Husbandry Company (Hezheng County, Gansu Province, China.). Eighteen healthy 2-year-old Simmental breeding cows (those that had calved within 7 days), with an average body weight of 550.0 ± 20 kg, were selected for the study. Randomized into two groups, 8 in the control (CON) group and 10 in the treatment (TRT) group, with a pre-feeding period of 10 d and an experimental period of 70 d. They were fed under the same phase, with 65% roughage (100% silage) + 35% concentrate in the diet of the control group (CON), and 65% roughage (50% maize straw pellet feed +50% silage) + 35% concentrate in the diet of the treatment group (TRT). The treatment group (TRT) was fed:65% roughage (50% corn stover pellets +50% silage) + 35% concentrate. Before the start of the trial, the barn was cleaned and disinfected, and the test cattle were dewormed and vaccinated, ear tagged, and stomach pumped with soda, and then the rations were put out at 7:00 and 17:00 every day during the trial period. At the end of the 70d period, milk samples, blood samples and rumen fluid samples were collected.

### 2.2 Diet formulation

The experimental diets were designed according to the Beef Cattle Feeding Standard’ (NY/T 815–2004) diet formulation, beef cattle nutritional recommendations for the standard formulation of different nutritional levels of concentrate and roughage pellet feed, roughage for Corn stover pellet feed (corn stover, alfalfa grass), silage, concentrate feed including maize, bran, rapeseed cake, concentrates, salt, premixed feeds, etc., and the composition of its diets and nutrient composition of the experimental rations The composition and nutrient content of the diets are shown in Table 1.

**Table 1:** Diet composition and nutritional composition (dry matter basis).

Items	CON	TRT
Diet composition		
Corn stalks/%	\	14.26
Alfalfa grass/%	\	6.20
Silage/%	83.48	57.01
Corn/%	10.99	15.02
Bran/%	1.68	2.30
Canola Cake/%	0.95	1.27
Concentrates/%	2.67	3.64
Premix additives /%	0.09	0.12
Salt/%	0.14	0.18
Total	100	100
Nutrient level		
Combined net energy (MJ/kg)*	49.70	59.93
Dry matter (DM, %)	87.44	91.53
Crude protein (CP, %)	10.58	11.97
Ether protein (EE, %)	3.91	2.14
Ash (%)	6.00	6.49
Neutral detergent fiber (NDF, %)	45.39	44.80
Acid detergent fiber (ADF, %)	29.34	27.12
Ca (%)	0.23	0.33
P (%)	0.30	0.33

Superscript * is calculated value, the rest is measured value.

### 2.3 Sample collection and data measurement

#### 2.3.1 Feed sample collection

About 500 g of each of the supplemented pelleted rations at different nutrient levels were taken in self-sealing bags and brought back to the laboratory for determination of each nutrient in the feeds. Straw pellets and silage samples were taken as 100 g each, oven dried at 105°C for 2 h, then baked at 65°C for 48 h to constant weight, removed to room temperature and weighed to calculate dry matter. The samples were crushed and sieved through a 40-mesh sieve for routine nutrient determination. Crude protein was determined by automatic Kjeldahl nitrogen tester; crude fat was extracted by Soxhlet extractor; acid washed fiber, neutral washed fiber and crude fiber were determined by fiber meter (Van Soest et al., 1991), crude ash was determined by burning at 550°C for 2 h in a muffle furnace and then weighed.

#### 2.3.2 Measurement of production performance

During the trial period (70 days), performance was evaluated based on a number of metrics including calving rate, calf survival, calf morbidity and breeding cows health. Observations were made by feedlot veterinarians and breeders on daily rounds, where calving rate was defined as the number of calves successfully delivered during the trial period as a percentage of the total number of breeding cows mated; calf survival was defined as the number of calves surviving within 70 days of birth as a percentage of the total number of calves born; calf morbidity was defined as the number of calves that were sick within 70 days of birth as a percentage of the total number of calves that survived at birth; and maternal health consisted of any observations made to assess the postpartum condition of the breeding cows. Maternal health status includes any health problems or complications observed to assess the postpartum condition of the breeding cows.

#### 2.3.3 Milk sample collection and measurement

At the end of the 70d experimental period, milk samples were collected from each breeding cows, divided into 50 mL freezing tubes (Breeding cows give milk for about 2 months), frozen and preserved to be brought back to the laboratory for the determination of milk composition, in which the content of milk fat rate, milk protein rate, lactose rate, milk protein and non-fat milk solids were determined using a Bulgarian (Lactoscan MCC500) fully automatic milk analyzer.

#### 2.3.4 Blood sample collection and measurement

At the end of 70d of the experimental period, blood was collected from the caudal vein of each breeding cows using a vacuum anticoagulation blood tube, and the collected blood samples were centrifuged at 3000r for 5 min in a freezing centrifuge after 30 min of resting and then the serum was separated, loaded into 2 mL freezing tubes, and stored at −80°C in an ultra-low-temperature refrigerator for the determination of the metabolism of the serum CON immune indexes.

Measurement of serum immunity indexes: Serum immunity kits were used to determine the serum levels of immunoglobulin A (IgA), immunoglobulin M (IgM), and immunoglobulin G (IgG), respectively; Serum metabolomics assay: Non-targeted serum metabolomics analysis was performed using a gas chromatograph (LC–MS; Agilent 1,290 Infinity LC ultra-high-pressure liquid chromatograph) for sample collection, metabolite extraction, QC preparation, sample LC–MS/MS, mass spectrometry, and data analysis. Pipette 100 μL serum sample in a 1.5 mL centrifuge tube, add 400 μL extraction solution (acetonitrile: methanol = 1:1) containing 0.02 mg/mL of internal standard (L-2-chlorophenylalanine), vortex mixing for 30 s, low temperature ultrasonication extraction for 30 min (5°C, 40 KHz), the sample static at −20°C for 30 min. 4°C, centrifugation at 13000 g for 15 The samples were extracted at −20°C for 30 min, centrifuged at 13000 g for 15 min at 4°C, and the supernatant was removed and blown dry under nitrogen. 100 μL of the compound solution (acetonitrile:water =1:1) was reconstituted, and then the samples were extracted by low-temperature ultrasonication for 5 min (5°C, 40 KHz), and the samples were centrifuged at 4°C for 10 min at 13,000 g, and then the supernatant was removed to the injection vials with the cannulae and analyzed on-line. An equal volume of all sample metabolites was mixed to prepare a quality control (QC) sample, and one QC sample was inserted every 5–15 samples during the instrumental analysis to examine the reproducibility of the whole analytical process. An equal volume of all sample metabolites were mixed and prepared as quality control (QC) samples, and one QC sample was inserted every 5–15 samples during instrumental analysis to examine the reproducibility of the whole analytical process. The samples were analyzed by LC–MS/MS on an ultra-high performance liquid chromatography tandem Fourier transform mass spectrometry UHPLC -Q Exactive HF-X system.

#### 2.3.5 Collection and measurement of rumen fluid

The rumen fluid was collected after 70d of the experimental period. A rumen catheter sampler was used for each breeding cows; the rumen tube was inserted into the oral cavity to the rumen, the rumen fluid was extracted by a syringe, and the former part of the effluent liquid was discarded before the pumping out, then the rumen fluid sample was collected, and the rumen fluid was filtered through gauze, the pH value was determined on the spot, and the rumen fluid was packed into 5 mL freezing storage tubes, and then divided into 5 portions and stored, and then put into liquid nitrogen and brought back to the laboratory for the determination of volatile fatty acid (VFA) and ammoniacal nitrogen, Rumen microbial diversity.

Ruminal fermentation parameters were determined: pH was measured by a pH (Starter 300, Shanghai, China) meter; volatile fatty acids (VFA), including acetic, propionic, butyric, valeric, and valeric acids, were determined in rumen liquor by gas chromatography (GC522, Shanghai, China; Erwin et al., 1961); and NH_3_-N concentration was determined by colorimetric method; 16S rRNA sequencing: The total genomic DNA of the microbial community was extracted according to the instructions of the E.Z.N.A.® soil DNA kit (Omega Bio-tek, Norcross, GA, United States), and the quality of the extracted genomic DNA was checked by agarose gel electrophoresis with 1% agarose, and the concentration and purity of the DNA were determined by NanoDrop2000 (Thermo Scientific, United States). The quality of the extracted genomic DNA was determined by 1% agarose gel electrophoresis, and the DNA concentration and purity were measured by NanoDrop2000 (Thermo Scientific, United States). The upstream primer 338F and the downstream primer 806R carrying the Barcode sequence were used for PCR amplification of the V3-V4 variable region of the 16S rRNA gene, and the amplification procedure was as follows: pre-denaturation at 95°C for 3 min, 27 cycles, followed by a stable extension at 72°C for 10 min, and then finally stored at 4°C. The PCR system was as follows: 5 × TransStart FastPfu buffer 4 μL, 2.5 mM dNTPs 2 μL, upstream primer (5uM) 0.8 μL, downstream primer (5uM) 0.8 μL, TransStart FastPfu DNA polymerase 0.4 μL, 10 ng of template DNA, and 20 μL. Three replicates were performed for each sample. PCR products from the same samples were mixed and recovered using a 2% agarose gel. The recovered products were purified using the AxyPrep DNA Gel Extraction Kit (Axygen Biosciences, Union City, CA, United States), detected by electrophoresis on a 2% agarose gel, and analyzed by Quantus™ Fluorometer (Promega, Inc., United States). The recovered products were purified by 2% agarose gel electrophoresis, detected and quantified by Quantus™ Fluorometer (Promega, United States).

### 2.4 Data analysis

The data of milk composition, serum immunity indexes, rumen pH, VFA and NH_3_-N of supplemented breeding cows were analyzed by one-way ANOVA using SPSS26.0, and the results were expressed as ‘mean ± standard deviation’, with *p*<0.05 as the criterion of significant difference.

Metabolomics data analysis: The raw data were converted into the. mzXML format by ProteoWizard, and then the XCMS program was used for peak alignment, Retention time correction and extraction of peak areas. The structure of metabolites was identified by exact mass number matching (< 25 ppm) and secondary spectrogram matching, and the laboratory self-established database was searched. For the data obtained from XCMS extraction, ion peaks with >50% missing values within the group were removed. The software SIMCA-P 14.1(Umetrics, Umea, Sweden) was used for pattern recognition. After Pareto-scaling preprocessing, multi-dimensional statistical analysis was performed, including unsupervised principal component analysis (PCA) analysis to evaluate the stability of the model. Supervised partial least squares discriminant analysis (PLS-DA) and orthogonal partial least squares discriminant analysis (OPLS-DA) which filters out signals that are not relevant to model classification and has explanatory power. The PLS-DA model was validated with 200 iterations and OPLS-DA displacement tests based on the Y (R2Y) modeling capability and model (Q2). When R2Y and Q2 indices are close to 1, the model is more stable and reliable. The Q2 regression line intercept was less than 0, and the model was not overfitted. In addition, Q2 > 0.5 indicates better predictive ability of the model. Unidimensional statistical analysis included Student’s t-test and fold change analysis, and R software was used to plot the volcano. Significantly different metabolites were screened based on variable weight values (VIP) obtained from the OPLS-DA model and Student’s t-test *p*-values. Metabolites with VIP >1 and *p* < 0.05 were considered differential metabolites. Pathways for differential metabolites were obtained by metabolic pathway annotation in KEGG database.

Analysis of 16S rRNA data: Ruminal microbial diversity analysis was performed using FLASHv1.2.7 software to overlap splice the reads of each sample to obtain raw Tags data (Raw Tags). Then, Raw Tags were filtered using Trimmomaticv0.33 software to obtain high quality Tags data (Clean Tags), and then chimeras were removed by UCHIME v4.2 software to finally obtain effective data (Effective Tags). Taxonomic analysis of OTU representative sequences (based on 97% similarity) was performed using RDP classifier and QIIME software. Alpha diversity analysis and dilution curve analysis were carried out in mothur platform. Venn diagrams were analyzed and plotted using R language, while LEfSe analysis analyzed the magnitude of differential effect by linear discriminant, with LDA > 4 as the basis for determination as the basis of judgment.

Descriptive statistics of serum metabolites, rumen fermentation parameters, and rumen microorganisms were performed using Microsoft Office Professional Plus Excel 2019 version. Correlation analysis of rumen microorganisms with serum metabolites and rumen microorganisms with rumen fermentation parameters was carried out using R (version 3.3.1) software. Correlation Heatmap analysis was carried out by calculating correlation coefficients (Spearman’s rank correlation coefficients, Pearson correlation coefficients, etc.) between the environmental factors and the selected species, and the matrix of values obtained was Visualization through Heatmap plots. Reflects the information of the data in a 2D matrix or table through color changes; the color shades indicate the magnitude of the data values; it visualizes the magnitude of the data values in defined color shades.

### 2.5 Technology roadmap

**Figure 1:**
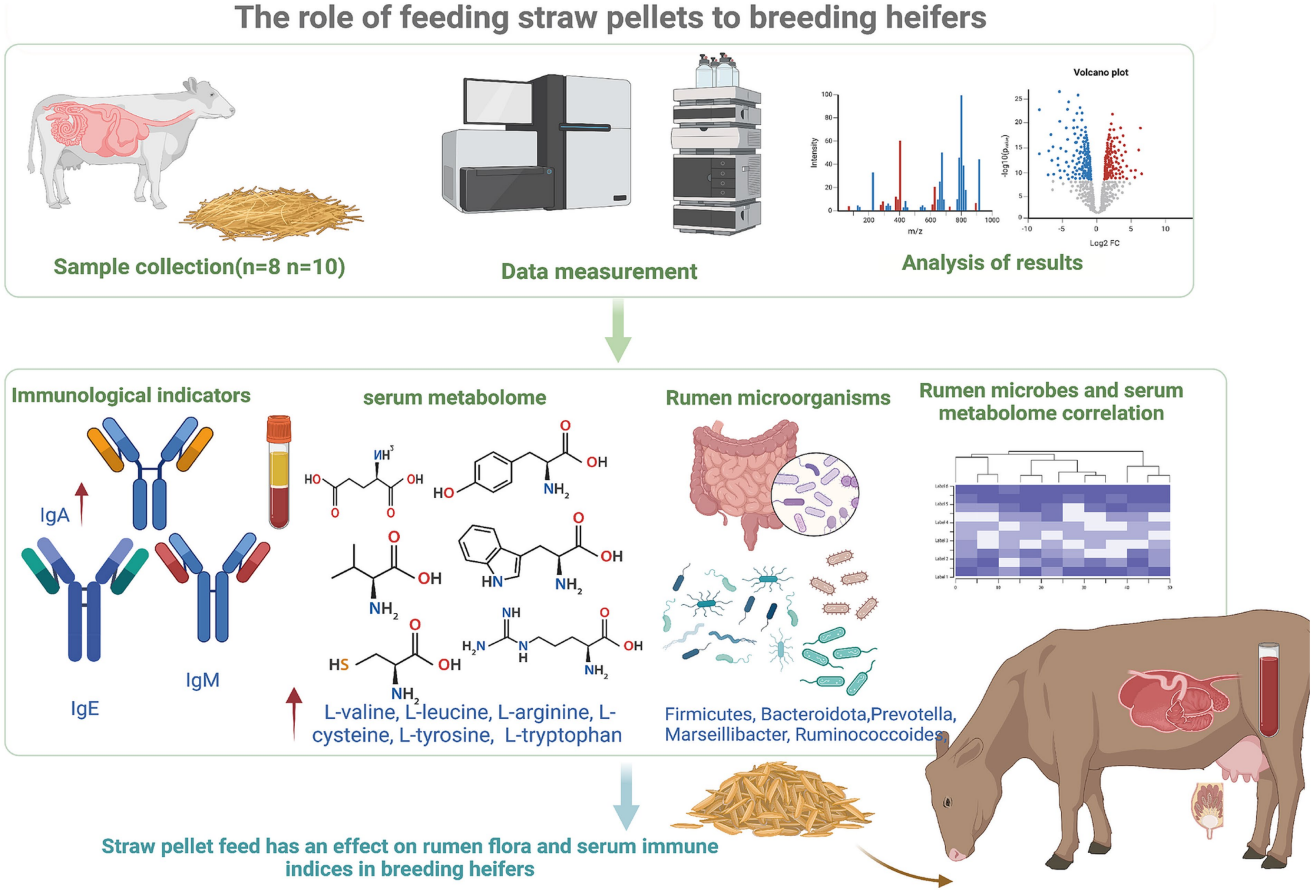
Technology roadmap.

As in Figure 1, the technical flowchart of this experiment.

## 3 Results

### 3.1 Effect of corn stover pellet feed replacing some silage on the performance of breeding cows

Straw pellet feed replacing part of silage on the production performance of breeding cows is shown in Table 2, in which there was no significant difference in the production performance of the CON and TRT groups (*p* < 0.05), indicating that corn stover pellet feed had no negative effect on the production performance of breeding cows.

**Table 2:** Production performance indicators.

Item	CON	TRT
Number of cows	8	10
Calving rate	100%	100%
Calf survival rate	100%	100%
Calving morbidity	0	0
Postpartum status of breeding cows	Favorable	Favorable

### 3.2 Effect of corn stover pellet feed replacing some silage on milk quality of breeding cows

Breeding cows milk quality can assess the nutritional value of milk and guide feeding management. The results of its straw pellet feed replacing part of silage on the milk composition of breeding ewes are shown in Table 3, the milk protein content and lactose content of the TRT group were significantly lower than that of the CON group (*p* < 0.05), and there was no significant difference in milk fat rate and total solids (*p* > 0.05).

**Table 3:** Milk composition analysis.

Item	CON	TRT	*p*-value
Fat%	3.353 ± 0.209	4.326 ± 1.338	0.060
Protein%	3.474 ± 0.128	2.908 ± 0.464	0.050
Lactose%	5.193 ± 0.189	4.362 ± 0.694	0.005
Total solids%	12.789 ± 0.477	12.243 ± 2.106	0.286

### 3.3 Effect of corn stover pellet feed replacing some silage on blood immunity indexes in breeding cows

Serum immunity assesses the status of the immune system and aids in the diagnosis of disease. The serum immunity indexes of breeding cows fed with straw pellets instead of some silage are shown in Table 4, in which immunoglobulin A (IgA) was significantly higher in the TRT group than in the CON group (*p* < 0.05), and there was no significant difference in immunoglobulin G (IgG) and immunoglobulin M (IgM) between the two groups (*p* > 0.05).

**Table 4:** Analysis of blood immunity indexes.

Item	CON	TRT	*p*-value
IgA g/l	2.292 ± 0.287	2.730 ± 0.525	0.04
IgG g/l	9.422 ± 1.001	10.567 ± 1.533	0.08
IgM g/l	0.685 ± 0.067	0.758 ± 0.114	0.13

### 3.4 Effect of corn stover pellet feed replacing some silage on blood metabolome in postpartum breeding cows

#### 3.4.1 Multivariate statistical analyses

**Figure 2:**
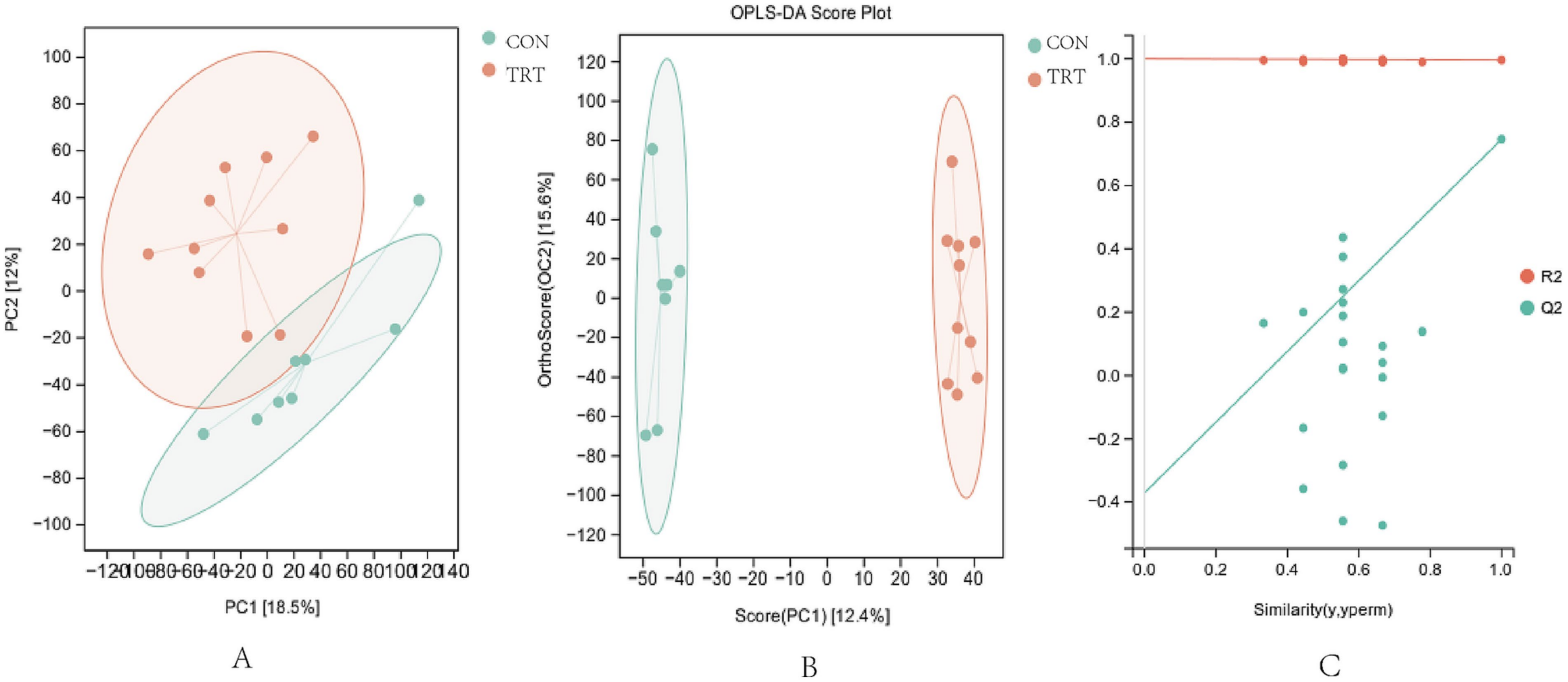
Multivariate statistical analysis chart. **(A)** Plot of PCA scores in positive ion mode. **(B)** OPLS-DA score chart in positive Ion mode. **(C)** OPLS-DA score chart in negative Ion mode.

**Figure 3:**
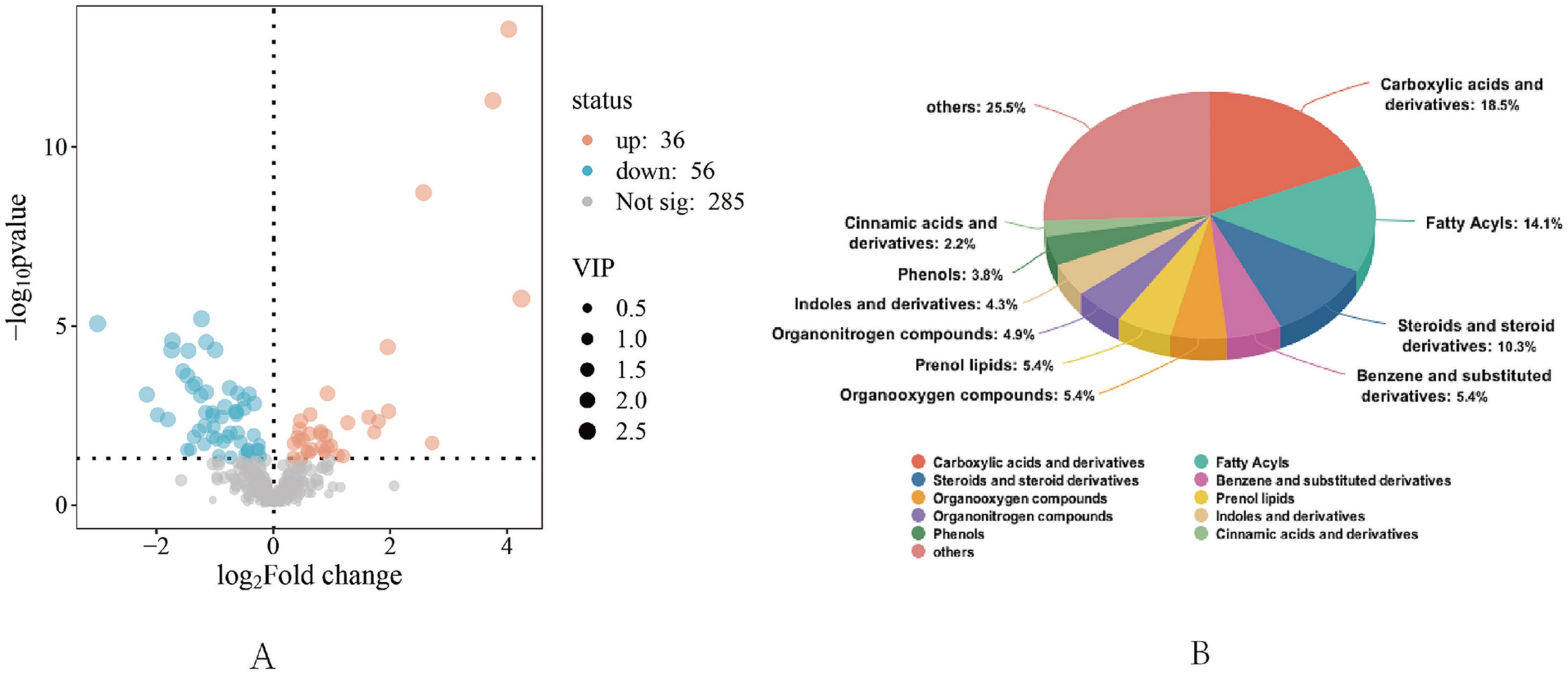
Differential metabolite screening and identification analysis chart. **(A)** General volcanic map, “Not Sig” represents metabolites with no significant difference (*p* > 0.05), “up” represents significantly up-regulated metabolites (*p* < 0.05), and “down” represents significantly down-regulated metabolites (*p* < 0.05). **(B)** Analysis of differential metabolite identification.

From the principal component analysis (PCA) score (Figure 2A), it can be seen that the metabolic components of t0he samples from groups CON and TRT showed a tendency to be partially separated in the positive ion mode, with significant separation occurring, indicating that there was a significant difference between the two groups (*p* < 0.05). The serum samples were further analyzed using orthogonal least partial squares discriminant analysis (OPLS-DA), Figure 2B, which showed significant separation and better aggregation. In order to test whether the OPLS DA model was overfitting, the OPLS-DA model was statistically validated by the replacement test, and the positive ion replacement test (R2Y = 0.996, Q2 = 0.746; Figure 2C), with R2Y close to 1 and the intercept less than 0,indicated that the model was robust and reliable, and overfitting did not occur, and the greater the degree of separation between the two groups of samples in the figure indicated that the more significant the classification effect was and that serum metabolite There was a significant difference between CON group and TRT group (*p* < 0.05; Figure 3).

#### 3.4.2 Differential metabolite screening

**Figure 4:**
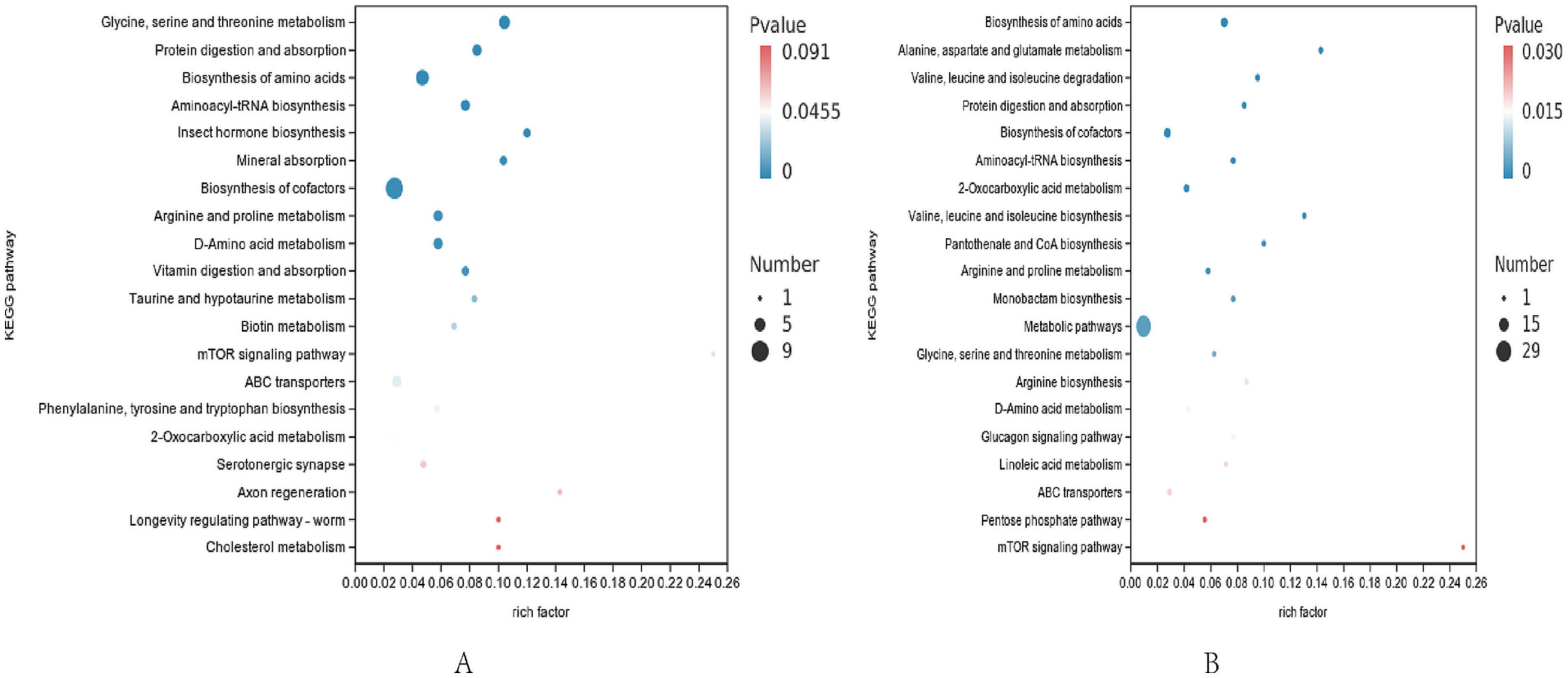
Differential metabolite pathway map. **(A)** Differential metabolite pathways in positive ion mode. **(B)** Differential metabolite pathways in negative ion mode. Rich factor refers to the ratio of the number of different metabolites enriched in the pathway to the number of different metabolites annotated, the larger the rich factor, the greater the enrichment.

A total of 92 differential metabolites were screened between groups in the positive and negative ion mode using VIP >1 and *p* < 0.05 as the standard, of which 36 expressed metabolites were up-regulated and 56 expressed metabolites were down-regulated. Among the metabolites with significant differences in serum, the visual analysis of the top 50 differential metabolites showed that L-valine, L-leucine, L-arginine, L-cysteine, L-tyrosine, and L-tryptophan were significantly up-regulated (*p* < 0.05; Figure 4).

The top 10 metabolites identified in total abundance in serum samples under different feeding regimens included carboxylic acids and derivatives, fatty acyls, steroids and steroid derivatives, benzene and substituted derivatives, organic oxides, isoprenoid lipids, organic nitrogen compounds, indoles and their derivatives, phenols, and cinnamic acid and its derivatives.

#### 3.4.3 Differential metabolite pathway analysis

Enrichment analysis of KEGG annotation results was performed to obtain the top 20 pathways enriched in differential metabolites. The positive ion pattern of differential metabolites in CON group and TRT group was mainly significantly enriched (*p* < 0.05) in six pathways in glycine, serine and threonine metabolism, protein digestion and absorption, mineral digestion and absorption, vitamin absorption, arginine and proline metabolism, phenylalanine, tyrosine, tryptophan biosynthesis and other pathways; the negative ion mode was mainly significantly enriched (*p* < 0.05) in amino acid biosynthesis, cofactor synthesis and so on.

### 3.5 Effect of corn stover pellet feed replacing some silage on rumen fermentation parameters in postpartum breeding cows

The results of rumen fermentation parameter measurements are shown in Table 5. There was no significant difference in rumen pH, rumen NH_3_-N content, and rumen acetic/propionic acid concentrations between CON group and TRT group (*p* > 0.05). Acetic acid, propionic acid, butyric acid, and total acid concentrations in CON group were significantly lower than those in TRT group (*p* < 0.05).

**Table 5:** Parameters of rumen fermentation in postpartum period of breeding cow fed with straw pellets instead of silage.

Item	CON	TRT	*p*-value
pH	6.854 ± 0.5174	6.613 ± 0.3075	0.276
NH_3_-N/mg·dL-1	29.90 ± 0.319	27.40 ± 0.255	0.865
Acetic/%	27.485 ± 6.185	38.394 ± 7.223	0.006
Propionic/%	7.647 ± 1.844	11.219 ± 2.540	0.006
Butyric/%	5.486 ± 1.998	7.43 ± 1.539	0.046
A/P	3.614 ± 0.297	3.483 ± 0.463	0.514
TVFA (mmol/L)	41.783 ± 10.11	58.31 ± 10.883	0.007

### 3.6 Effect of corn stover pellet feed replacing some silage on rumen microorganisms in postpartum breeding cows

#### 3.6.1 Rumen microbial diversity and abundance

**Figure 5:**
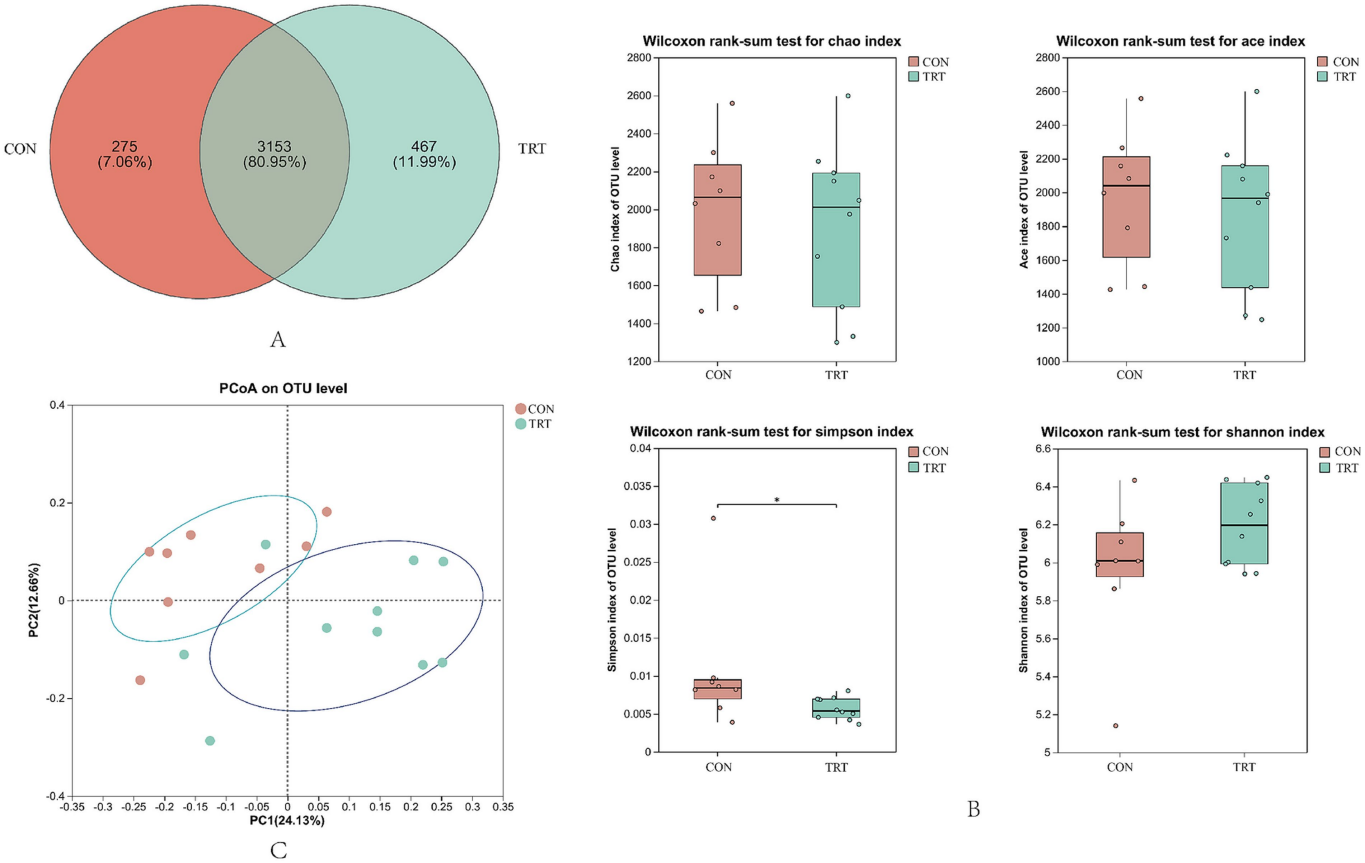
Rumen microbial diversity and abundance maps. **(A)** Venn diagram of rumen flora diversity. **(B)** Analysis of alpha diversity. **(C)** PCoA analysis.

The Venn diagram of rumen flora diversity is shown in Figure 5A, sharing 3,153 OTUs, whereas groups CON group and TRT group had 275 and 467 exclusive OTUs, respectively.

Alpha diversity index reflected the community richness and diversity of gastrointestinal flora, in which there was no significant difference in Ace index, Chao1 index, Shannon index (*p* > 0.05), and significant difference in Simpson index (*p* < 0.05; Figure 5B). Beta diversity was mainly used to study the structural differences of microbial flora in different samples or groups and to judge by the proximity of the sample sites Individual or intergroup flora differences. Rumen microbial communities of breeding cows in CON group and TRT group were subjected to PcoA analysis, and the principal component contribution values of PC1 were 24.13%, and PC2 was 12.66%, as shown in Figure 5C, with individual samples relatively close to each other between the two groups.

#### 3.6.2 Composition of rumen flora at phylum and genus taxonomic levels

At the phylum classification level, the top 10 dominant rumen flora in terms of relative abundance were, in descending order, Firmicutes, Bacteroidota, Mycoplasmatota, Planctomycetota, Spirochaetota, Proteobacteria, Chloroflexota, Actinomycetota, Thermodesulfobacteriota, Kiritimatiellota.

At the genus taxonomic level, the top 10 dominant rumen flora in terms of relative abundance were in the order of *Prevotella, Marseillibacter, Ruminococcoides, Acetivibrio, Intestinimonas, Spiroplasma, Saccharofermentans, Luoshenia, Eubacterium, Butyrivibrio*.

#### 3.6.3 Differences in relative abundance of rumen bacteria

**Figure 6:**
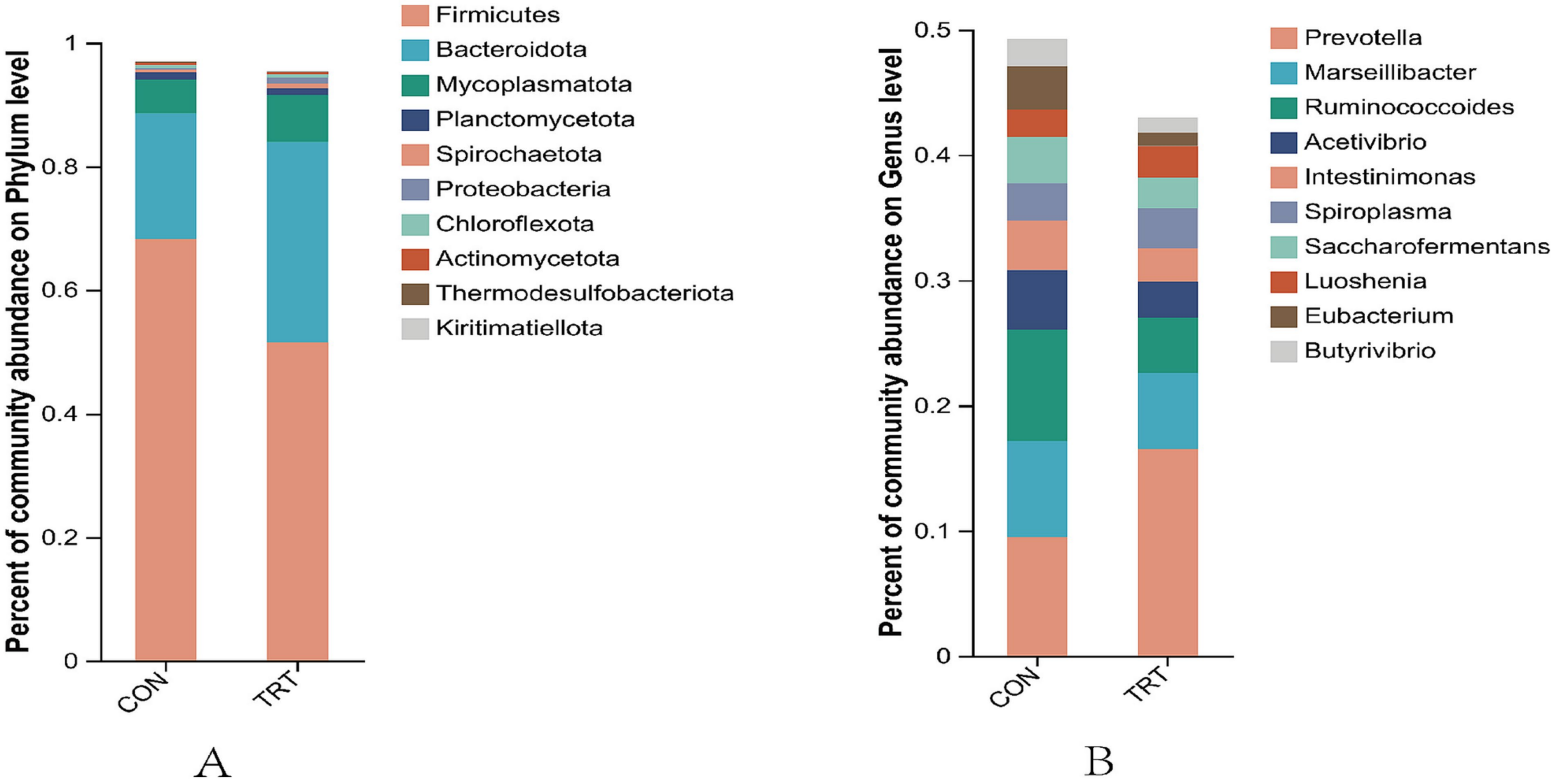
Diagram of the taxonomic level of phylums and genera. **(A)** Distribution at the phylum level. **(B)** Distribution of communities at the taxonomic level.

Intergroup Wilcox rank sum test for rumen microbiology of breeding cows under both feeding regimens was performed for species with differing results. At the level of Firmicutes was significantly higher in CON than in TRT (*p* < 0.05), and the Bacteroidota, Mycoplasmatota, and Proteobacteria were significantly higher in TRT group than in CON group (*p* < 0.05; Figure 6).

**Figure 7:**
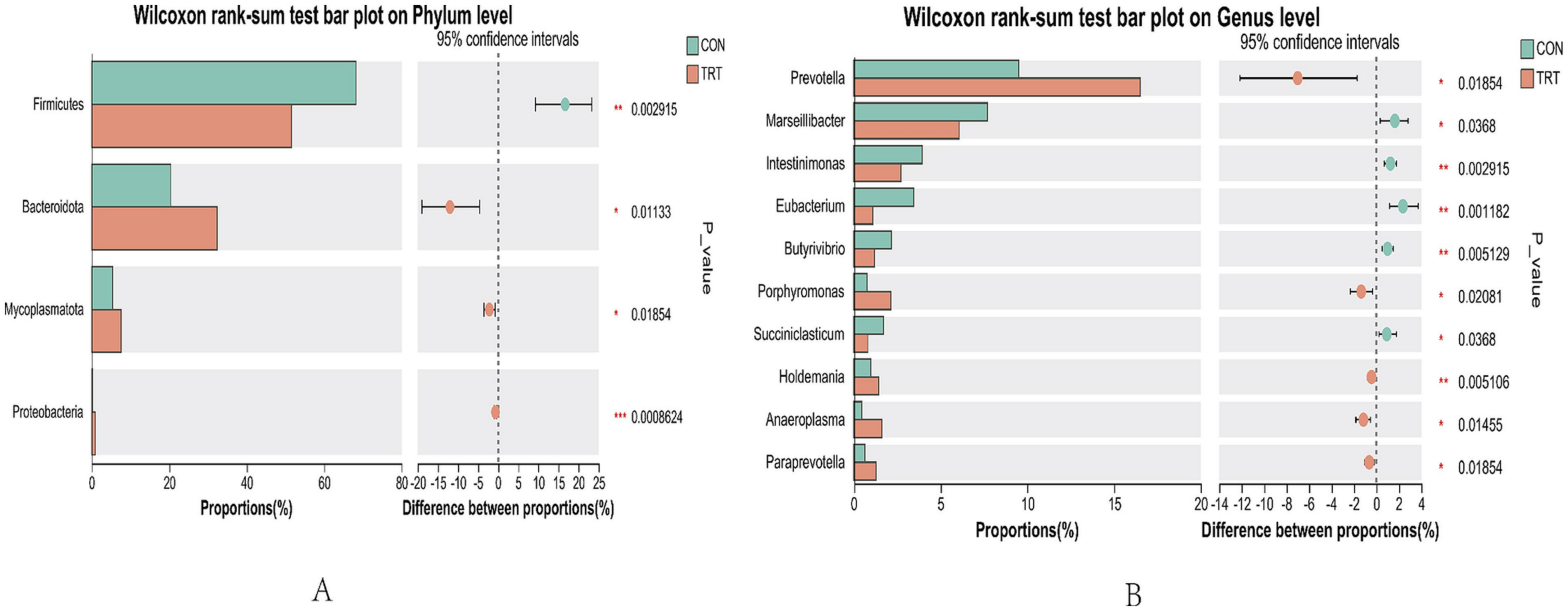
Plot of differences in relative abundance of rumen bacteria. **(A)** analysis of differences in flora at the level of phylum. **(B)** Analysis of differences in flora at the level of genus.

Analyzing differences in taxonomic levels of genera, TRT was found to have a significantly higher abundance of *Prevotella*, *Porphyromonas, Holdemania, Anaeroplasma*, and *Paraprevotella* than CON group (*p* < 0.05). The abundance of *Marseillibacter, Intestinimonas, Eubacterium, Butyrivibrio, and Succiniclasticum* was significantly higher in CON group than in TRT group (*p* < 0.05; Figure 7).

#### 3.6.4 Differential analysis of ruminal flora

**Figure 8:**
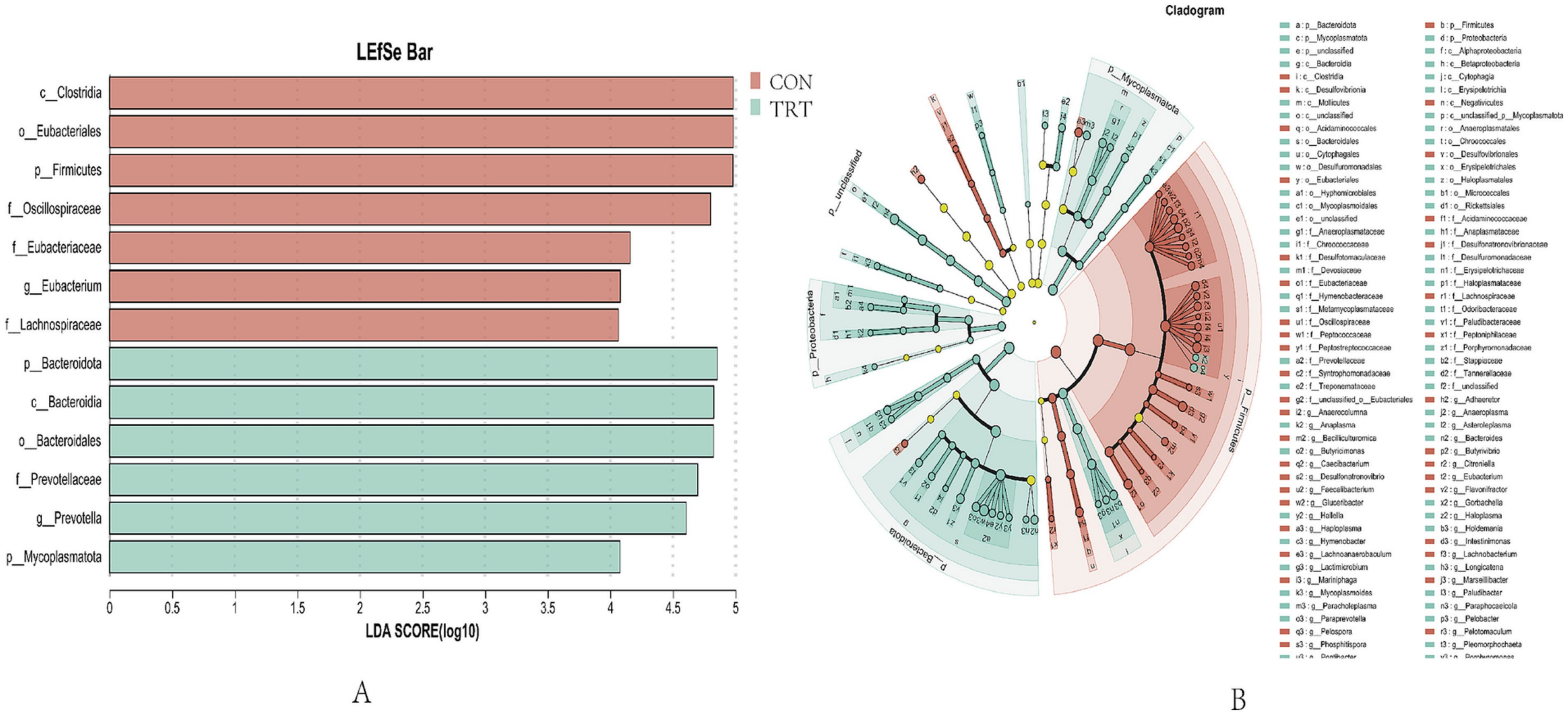
Similarity maps of rumen microbial communities. **(A)** LEfse discriminant analysis for rumen flora characterization (LDA>4). **(B)** LEfSe multilevel species hierarchical tree map (LDA>2).

Based on the above analysis of the diversity, composition and similarity of rumen microbial communities in breeding cows, biomarkers of rumen microbial communities in breeding cows fed different levels of rations were screened by linear discriminant analysis (LEfSe). LefSe plots include multilevel species hierarchical tree plots and histograms of LDA value distributions to analyze differences in microbi-al composition and obtain species with significant differences. As shown in Figure 8A, the LEfSe analysis (LDA threshold >4) showed that the bacteria in the rumen remodeled as the nutrient level of the ratio changed. As shown in Figure 8B, by LEfSe analysis (LDA threshold >2) where there were 38 biomarkers in the CON group and 62 biometabolites in the TRT group.

### 3.7 Analysis of rumen fermentation parameters and microbiological correlations, rumen microbiological and serum metabolomic correlations in postpartum rumen fermentation in breeding cows on straw pellets replacing part of silage

**Figure 9:**
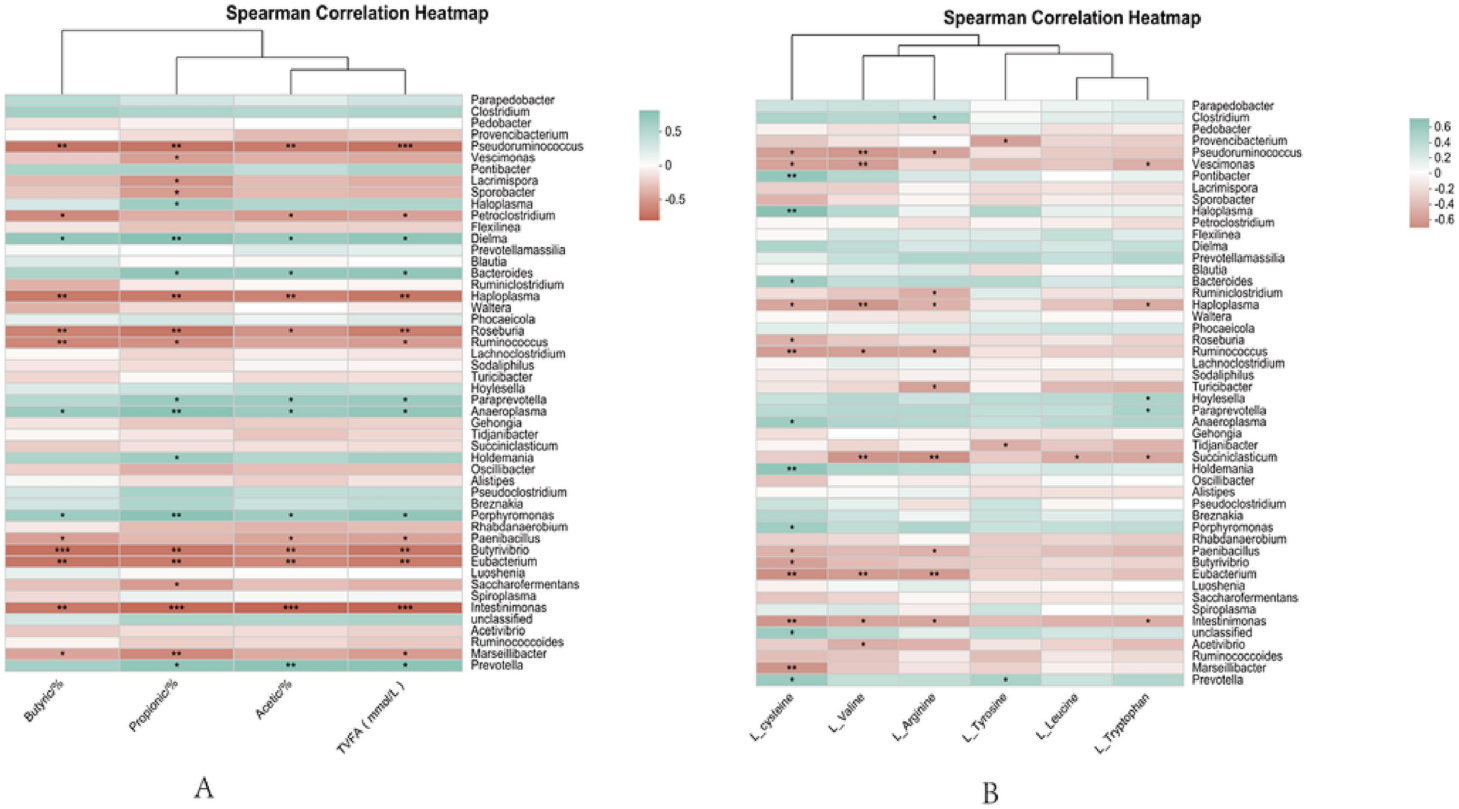
Correlation analysis chart. **(A)** Analysis of rumen fermentation parameters and microbiological correlations. **(B)** Rumen microbial and serum metabolite correlation plots; *0.01; *p* ≤ 0.05, **0.001; *p* ≤ 0.01.

As seen in Figure 9A, propionic and TVFA were significantly positively correlated with *Prevotella* (*p* < 0.05); butyric was highly significantly positively correlated with *Prevotella* (*p* < 0.01); Propionic acid, butyric acid, and total acid were positively correlated (*p* < 0.05) with *Bacteroides*; Acetic, propionic, butyric, and TVFA showed highly significant negative correlation (*p* < 0.01) with *Eubaterium*.

As seen in Figure 9B, L-cysteine showed a significant positive correlation with *Prevotella* and *Anaerobicum* (*p* < 0.05) and a highly significant positive correlation with *Eubacterium* (*p* < 0.01), Significantly negatively correlated with *Bacteroides* (*p* < 0.05) Very significantly negatively correlated with *Ruminalococcus* (*p* < 0.01); L-valine showed a significant negative correlation with *Acetivibrio* (*p* < 0.05) and a highly significant negative correlation with *Eubaterium* and *Succiniclasticum* (*p* < 0.01); L-arginine showed highly significant negative correlation (*p* < 0.01) with *Eubaterium* and *Succiniclasticum*; L-tyrosine was significantly and positively correlated with *Prevotella* (*p* < 0.05); L-leucine was significantly (*p* < 0.05) negatively correlated with *Succiniclasticum*; L-tryptophan showed a significant negative correlation with *Succiniclasticum* (*p* < 0.05) and a significant positive correlation with *Holdemania* (*p* < 0.05).

## 4 Discussion

Milk fat and milk protein content are important indicators of milk quality, and milk fat is closely related to physiological processes such as early growth and development and immune regulation in animals (McManaman, 2009). Chiou et al. (1998) suggested that the level of milk protein rate is related to the energy intake in the ration, and lactose is the main carbohydrate in dairy products and maintains the quality of dairy products (Hemmler et al., 2018). In this experiment, the content of milk fat and total solids in the TRT group did not change significantly (*p* > 0.05) compared to the CON group, and the content of lactose and milk protein in the TRT group decreased significantly (*p* < 0.05) compared to the CON group. In the study of milk quality of Simmental breeding cows (Koç and Öner, 2023), the contents of the milk components were in agreement with the basic results of this pilot study, and the milk quality content was within the normal range. This shows that replacing part of the silage with corn stover pellets has no adverse effect on milk quality and ensures the healthy growth of the breeding cows.

Immunoglobulin is the main component of humoral immunity in animals, and there is a large amount of immunoglobulin in the serum, which has a variety of functions such as antimicrobial and antivirus (Chen et al., 1994). Immunoglobulins, including IgA, IgG, and IgM, are produced by B cells during activation and differentiation in lymph nodes in response to antigens or allergens and are an important component of the host’s humoral immunity against pathogens and viruses (Shamji et al., 2021). IgA has a stronger and longer-lasting effect in neutralizing viruses than IgG (Sterlin et al., 2021). IgA is responsive to specific bacteria and viruses in addition to non-specific types of pathogens (Sugino et al., 2021). In the gut, IgA contributes to the establishment of a reciprocal host-microbiota relationship, thereby maintaining homeostasis and preventing disease (Chen et al., 2020). Van Hoeij et al. studied that protein-rich diets boosted the immune function of dairy breeding cows, reduced the body’s inflammatory response, and made them more resistant to disease (van Hoeij et al., 2017). Studies have shown that acetic acid induces IgA production by modulating the interaction between intestinal epithelial cells and immune cells, and that serum levels of IgA are increasing, resulting in the maintenance of higher levels of immunity (Takeuchi et al., 2021). In this experimental study, it was found that the number of immunoglobulin A in the serum of ewes in the straw pellet feeding group increased, and the immune response ability was enhanced, which may be due to the high fiber content in straw pellets, which promotes intestinal peristalsis, and the fiber has an adsorption effect, which can adsorb toxic substances in the intestinal tract, reduce the disease and enhance immunity, and reduce the inflammatory reaction of the organism.

A stable intra-rumen environment is essential for the health of ruminants, and rumen pH, ammonia-nitrogen (NH_3_-N) concentration and volatile fatty acid (VFA) concentration are key indicators for assessing the stability of the rumen environment, which can reflect the state of rumen fermentation (Tomczak et al., 2019). The results of Wang et al. (2020) showed that changes in the nutritional level of beef cattle diets can affect the composition and activity of rumen microorganisms, which in turn can alter rumen pH and influence the metabolic regulation of serum short-chain fatty acids. Changes in dietary nutrient levels are the main factors leading to fluctuations in rumen pH. In this experiment, the rumen pH of the breeding cows decreased, which may be due to the acidic species contained in the straw itself, and the dissolution of acids during the process of making pellets lowered the pH, but the pH was within the normal range, which indicated that the feeding of the groups was relatively stable and did not adversely affect the internal environment of the rumen. Higher concentrations of ammonia nitrogen usually contribute to the synthesis of microbial proteins (Lara et al., 2018; Rabelo et al., 2018), and studies have shown that microorganisms in the rumen can convert NH_3_-N into microbial proteins, a process that is directly influenced by the availability of fermentable carbohydrates (Zurak et al., 2023). No significant differences in rumen fluid NH_3_-N concentrations were observed between the two groups in this study. The rumen’s self-regulatory capacity ensured relatively stable protein and non-protein nitrogen metabolism rates despite the different dietary energy levels. Continuous provision of crude protein in diets fed at different energy levels ensured continuous utilization of nitrogen sources by rumen microorganisms. This contributed to maintaining a stable concentration of ammonia nitrogen NH_3_-N in the rumen. Nutritional level of the ration has a significant effect on the rumen fermentation process in breeding cows, and proper ration formulation is essential to maintain rumen function; type of diet and nutritional level affect VFA production (Rabelo et al., 2016). VFA also play an important role in increasing apparent dry matter digestibility and microbial growth as well as enhancing microbial function and enzyme activity (Apajalahti et al., 2019). VFA is a major product of rumen microbiota fermentation, and VFA concentration may reflect changes in rumen fermentation patterns (Reis et al., 2016; Beckett et al., 2021). The ruminal TVFA (total volatile fatty acid) concentration in the straw pellet group was significantly higher than that in the silage group in this study, and the concentration of ruminal TVFAs increased significantly with increasing dietary protein level, which is consistent with the results of studies on buffaloes and yaks (He et al., 2024; Wang et al., 2024). With a corresponding increase in acetic acid concentration at increased crude fiber content, while propionic acid and butyric acid concentrations are highly correlated with non-fiber carbohydrates (NFC) in the diet (Kaufmann and Rohr, 1966). Acetic acid is the main precursor for fat synthesis, while propionic acid mainly promotes glucose synthesis. Acetic acid production is more dependent on fiber fermentation, and fiber-degrading bacteria in the rumen can utilize carbohydrates to produce acetic acid, and the higher relative abundance of fiber-degrading bacteria in the straw pellet group further confirms the variation in acetic acid concentration (Hristov and Ropp, 2003). Since acetic acid constitutes a large proportion of total VFA, an increase in acetate content is accompanied by an increase in total VFA content. According to Cui et al. (2023) an increase in the concentration of propionic acid in the rumen indicates an improvement in the efficiency of energy conversion, a process that is important for the weight gain of animals. Propionic acid is essential for glucose synthesis in the body, and higher levels will enhance the availability of energy for body growth (Kristensen and Harmon, 2004; Askar et al., 2023). Increased rumen TVFA concentrations promoted the degradation of cellulose in the diet, suggesting a more balanced diet under this feeding condition, which improves nutrient digestibility and influences rumen microbial composition, and consequently rumen fermentation.

It has been shown that the composition of the host’s diet and its own growth and development also affect the diversity of bacteria in the gut (Ley et al., 2008). Chanthakhoun et al. (2012) found that different types of roughage fed to beef cattle can change the rumen flora, of which Firmicutes and Bacteroidetes are the dominant rumen flora and play an important role in rumen fermentation (Singh et al., 2012; de Oliveira et al., 2013). The Firmicutes contributes to the efficiency of cellulose breakdown and utilization in feeds (Iljazovic et al., 2021), while the Bacteroidetes is involved in nutrient metabolism processes such as carbohydrate fermentation and polysaccharide metabolism (Li et al., 2021). The dominant phylum in breeding cows’ rumen coincided with results from previous studies. The proportions of Firmicutes and Bacteroidetes in the CON and TRT groups in this experiment were 68.20 and 20.4%, 51.53 and 32.41%, respectively. The proportions of Firmicutes and Bacteroidetes were high in both groups. The relative abundance of Bacteroidetes was significantly higher in the TRT group than in the CON group, indicating that feeding corn stover-based diets could help to promote the proliferation of Bacteroidetes and thus improve the utilization efficiency of nutrients such as cellulose and polysaccharides (Petri et al., 2013), and the change of fiber structure improved the intra-rumen environment, which was more suitable for the survival of Bacteroidetes, indicating that roughage feeding to beef cattle can change the rumen flora, which is consistent with the results of the above study. Feeding diets with different nutrient levels resulted in changes in the abundance and structure of the rumen flora; Henderson et al. studied the rumen microbiota of 32 species, including buffalo, bison, and sheep, and found that dietary changes resulted in changes in rumen microbial genera (Henderson et al., 2015). The main genera in this experiment are *Prevotell* and *Ruminococcoides*. *Prevotella* play an important role in maintaining microecological balance in the digestive tract (Daniel et al., 2013). The number of rumen bacteria can reach 60–70% of the total rumen flora, although it does not degrade cellulose, it is one of the main protein degrading bacteria in the rumen, and it is crucial for the degradation of starch, hemicellulose, lignin, and pectin (Avgustin et al., 1997). Pitta et al. showed a significant increase in rumen *Prevotella* spp. in beef cattle fed wheat straw high in crude protein (Pitta et al., 2014). A study showing higher rumen *Prevotella* spp. abundance in goats fed high forages (Grilli et al., 2016). The relative abundance of *Prevotella* increased with the addition of straw pellets to the ration in this experiment, which is consistent with the results of previous studies, suggesting that an increase in protein concentration leads to a high number of *Prevotella* and that *Prevotella* may also enhance the degradation of starch, hemicellulose, lignin, and pectin by breeding cows. Meanwhile, the fermentation products of *Prevotella*in the rumen are mainly acetic, propionic, and succinic acids, while succinic acid is quickly converted to propionic acid by microbial enzymes (Pope et al., 2011). The relative abundance of *Prevotella* was significantly positively correlated with propionic acid with an increase in the proportion of Prevotella indicating that *Prevotella* were closely related to the metabolism of VFA in the rumen. *Ruminococcoides*, on the other hand, are rumen microorganisms that produce acetic, formic and succinic acids by breaking down plant fibers (Henderson et al., 2016). Elevated abundance may improve feed conversion rates, and breeding cows fed silage in this study had higher abundance of rumenococci. This may be attributed to the high content of silage in the diet and the partial breakdown of nutrients during fermentation, which provides a good nutrient base for growth and reproduction of rumenococci. The increased abundance of rumen microorganisms may have been accompanied by higher levels of propionic, butyric and total acids, further supporting the effect of feeding different types of feeds on the microbial community.

Using LC–MS studies to analyze changes in the serum metabolome of breeding cows after parturition, the metabolomic data showed that different feed types altered the concentrations of serum metabolites in the blood and suggested that serum metabolism may be related to rumen microbiota activity, and found that feed type altered the concentrations of most metabolites associated with amino acid metabolism. Whereas energy metabolism is a complex process of producing energy from nutrients and includes interrelated pathways such as lipid and amino acid metabolism (Velayudhan et al., 2015; Chen and Zhang, 2021). Amino acids are nutritional substrates for protein synthesis and also participate in nutrient metabolism as biologically active molecules (Tsukano and Suzuki, 2019). Among them, arginine is a semi-essential amino acid in most mammals, including humans and rats, and studies have found that dietary supplementation of arginine or its intermediate products, such as citrulline during pregnancy, can promote fetal growth in rats, piglets, and ewes (Bourdon et al., 2016; Wu et al., 2013; Tain et al., 2010). L-Arginine (L-Arg) is a substrate for the synthesis of nitric oxide, which activates soluble guanylate cyclase to promote vasodilation and induce insulin release (Meneguello et al., 2003), has also been shown to be involved in the TCA cycle by influencing fumarate formation (Sugiyama et al., 2014). Jiao et al. also noted that arginine in the urea cycle is associated with the synthesis of antioxidant and anti-inflammatory molecules (Jiao et al., 2015). In this study, L-Arg metabolites were up-regulated in the stover pellet group, which is mainly due to the fact that corn stover pellet feed is a cereal that may contain arginine itself, and some of the flora can also synthesize arginine and absorb it into the serum through the intestine, and it is hypothesized that feeding stover pellet feed can regulate oxidative stress and have a positive effect on arginine enhancement. L-Cysteine (L-Cys) is a natural amino acid with important physiological and pharmacological functions (Iyer et al., 2009). Mammalian livers generally have a pool of free cysteine, and intracellular cysteine content can be regulated to maintain at a certain level regardless of insufficient or excessive intake of sulfur-containing amino acids in the body Mammalian livers generally have a pool of free cysteine, and intracellular cysteine content can be regulated to maintain at a certain level regardless of insufficient or excessive intake of sulfur-containing amino acids in the body (Stipanuk et al., 2006). It is both a raw material for the synthesis of glutathione in plasma erythrocytes and a soluble antioxidant that maintains the proper intracellular and extracellular redox state (Yildiz et al., 2006). L-Cys was up-regulated in the straw pellet group and positively correlated with *Prevotella*. These are common microorganisms in the rumen that can utilize L-Cys as part of their metabolism to promote their growth and reproduction, which can influence the structure of the microbial community, with implications for the health and performance of cows. L-Cys is positively correlated with the presentation of anaerobic *Anearoplasm*. It is anaerobic bacteria and anaerobic fungi produce multi-enzyme complexes called cellulosomes that enable them to rapidly extract sugars from recalcitrant plant biomass, and rumen fungi secrete a wide range of degrading enzymes throughout their growth and development, including a variety of fiber-degrading enzymes, which are essential for the digestion and utilization of plant fibers in ruminants (Fontes and Gilbert, 2010). Anaerobic bacteria significantly impact ruminant diets, and L-Cys provides essential nutrients to the flora for normal physiological value-added. L-tyrosine (L-Tyr) is an important amino acid, and L-Tyr metabolism may provide certain metabolites or sources of nitrogen required for microbial growth. Tryptophan (Trp) is an important substrate involved in protein synthesis in living organisms and is mainly derived from dietary (Kałużna-Czaplińska et al., 2019). Jansman et al. found a strong positive correlation between tryptophan (Trp) concentration and the rate of protein synthesis (Kim et al., 2010), as well as an increase in energy synthesis by stimulating insulin secretion and activating translation initiation factors to initiate translation (Adeola and Ball, 1992). L-Trp reduces animal consumption, and its catabolism promotes the production of antimicrobial peptides to attenuate intestinal inflammatory responses and modulate intestinal immune tolerance. The up-regulation of L-Trp in the straw pellet group in this experiment was closely related to the increase in serum immunity indexes we studied, and the increase in L-Trp could enhance immunity. It has been shown that cows fed corn concentrates have increased serum levels of both tyrosine and tryptophan, with sufficient substrate levels for protein synthesis and synthesis rates (Chen et al., 2021), which is consistent with the results of this study. *Holdemania* in the digestive process, which degrades complex carbohydrates, L-Trp was positively correlated with *Holdemania*, and it was hypothesized that straw pellet feed *Holdemania* promotes L-Trp synthesis, and it was thought that the changes in rumen micro may be related to amino acid metabolism of serum and bacterial composition are closely related. L-Valine (L-Val) is one of the essential amino acids with multiple biological roles in regulating protein synthesis, lipid metabolism, glucose metabolism, antioxidant defense, and immune functions (Walejko et al., 2021; Lee et al., 2019). Val is also an important signaling factor that activates the mTOR complex pathway and regulates insulin sensitivity, thereby regulating tissue protein synthesis and energy metabolism in ruminants (Appuhamy et al., 2012; Toerien et al., 2010). L-Val is a crucial gluconeogenic amino acid in the cow’s organism and is closely related to production performance (Hultquist and Casper, 2016). Some studies have shown that Val injection can enhance the immune function of mice and boost the pathogen phagocytosis of macrophages (Chen X. H. et al., 2017). L-Val levels were upregulated, which could explain the elevated protein retention and enhanced antioxidant and immunity. L-Leucine (L-Leu) is a branched-chain amino acid involved in energy and muscle metabolism, with the metabolic end-products being acetyl coenzyme A and acetoacetate (Zhao et al., 2024), L-Leu also stimulates insulin release and promotes protein biosynthesis (Etzel, 2004). Straw pellet diets were up-regulated in L-Leu, indicating that dietary changes can promote protein synthesis. *Succiniclasticum*, a genus that plays a crucial role in the core rumen microbiota, is responsible for the degradation of carbohydrates to succinic acid and continues to participate in the metabolic process that leads to the production of propionic acid (Wang et al., 2021). L-Arg, L-Val, L-Trp, and L-Leu were significantly negatively correlated with Succiniclasticum, which may be due to the preferential utilization of these amino acids by other microorganisms or competitors of *Succiniclasticum*, decreasing the availability to *Succiniclasticum* and thus inhibiting the growth of these flora. The up-regulation of amino acid differential metabolites suggests that feeding straw pellets to breeding cows is beneficial, promotes the production of favorable metabolites in the serum of breeding cows, helps the body to regulate amino acid metabolism to maintain metabolic adaptations in the internal environment and enhances the immune competence, which in turn reduces the disruption of the serum barrier function.

## 5 Conclusion

In this study, microbiome and metabolomics analyses were combined to investigate the association between specific flora and serum metabolites in the rumen of breeding cows, which were significantly affected by different feeds, and also regulate the rumen flora structure, improve serum immune indexes, and save feed costs. The substitution of straw pellets can better affect the postpartum occurrence of breeding cows, and the relationship between microorganisms and metabolites has been further investigated. It also lays the foundation for future scientific feeding of breeding cows.

## Data Availability

The microbial sequencing data provided in this study can be found in the GenBank Sequence Read Archive (SRA) database under accession number PRJNA1166757.

## Glossary

CON: Control group
TRT: Treatment group
IgA: Immunoglobulin A
IgG: Immunoglobulin G
IgM: Immunoglobulin M
VFA: Volatile fatty acids
TVFA: Total volatile fatty acids
LC-MS: Liquid Chromatograph Mass Spectrometer
16S rRNA: 16S ribosomal RNA
QC: quality control
DM: Dry matter
CP: Crude protein
EE: Ether protein
NDF: Neutral detergent fibe
ADF: Acid detergent fiber
VIP: Variable Importance in Projection
PCA: principal component analysis
LDA: linear discriminant analysis
OTU: Operational Taxonomic Unit
LEfSe: Linear discriminant analysis Effect Size
OPLS-DA: Orthogonal Partial Least Squares Discriminant Analysis
L-Cys: L-Cysteine
L-Tyr: L-tyrosine
Trp: Tryptophan
L-Trp: L-Tryptophan
L-Val: L-Valine
